# Outcomes for Sinonasal Undifferentiated Carcinoma (SNUC): An International Multi-Center Retrospective Cohort Study

**DOI:** 10.3390/cancers18030366

**Published:** 2026-01-24

**Authors:** Jacklyn Liu, Yoko Takahashi, Umar Rehman, Mario Turri-Zanoni, Davide Mattavelli, Nicholas Counsell, Marco Ferrari, Vittorio Rampinelli, William Vermi, Davide Lombardi, Rami Saade, Ki Wan Park, Oscar Emanuel, Volker H. Schartinger, Alessandro Franchi, Carla Facco, Fausto Sessa, Simonetta Battocchio, Patrick Rene Gerhard Eriksen, Simone Kloch Bendtsen, Kathrine Kronberg Jakobsen, Mohamed el Haddouchi, Roberta Maragliano, Giedrius Lelkaitis, Anirudh Saraswathula, Raman Preet Kaur, Wojciech K. Mydlarz, Murugappan Ramanathan, Masaru Ishii, Manas Dave, Tim R. Fenton, Alison Lim, Saleh Okhovat, Gyleen Elegio, Charles Dupin, Pierre Pouvreau, Juliette Thariat, Laurence Digue, Francois-Regis Ferrand, Valerie Costes-Martineau, Claire Castain, Héloïse De Kermadec, Justin Hintze, James Paul O’Neill, Peter Lacy, Francis M. Vaz, Paul O’Flynn, David J. Howard, Paul Stimpson, Simon Wang, Gary Royle, Christopher Steele, Amrita Jay, Dawn Carnell, Martin D. Forster, David Thomson, Christian von Buchwald, Robbie Woods, Jose Luis Lllorente, Mario Hermsen, Philipp Jurmeister, David Capper, Gary L. Gallia, Joshua K. Tay, Ahmed Mohyeldin, Juan Fernandez-Miranda, Quynh-Thu Le, Robert B. West, Zara M. Patel, Jayakar V. Nayak, Peter H. Hwang, Fabio Facchetti, Piero Nicolai, Renata Ferrarotto, Jack Phan, Paolo Bossi, Paolo Castelnuovo, Antoine Moya-Plana, Benjamin Verillaud, Cathie Garnis, Andrew Thamboo, Felicia Olawuni, Eric J. Moore, Garret Choby, Devyani Lal, Neal Akhave, Diana Bell, Shirley Y. Su, Valerie J. Lund, Nyall R. London, Ehab Y. Hanna, Matt Lechner

**Affiliations:** 1 Division of Surgery and Interventional Science, University College London, 43-45 Foley St, London W1W 7TY, UK; jih.liu.18@ucl.ac.uk (J.L.); u.rehman@ucl.ac.uk (U.R.); oscar.emanuel@nhs.net (O.E.); 2 UCL Cancer Institute, University College London, Paul O’Gorman Building, 72 Huntley St, London WC1E 6DD, UK; g.royle@ucl.ac.uk (G.R.); c.steele.11@ucl.ac.uk (C.S.); m.forster@ucl.ac.uk (M.D.F.); 3 Department of Head and Neck Surgery, The University of Texas MD Anderson Cancer Center, 1515 Holcombe Boulevard, Houston, TX 77030, USA; ytakahas@mdanderson.org (Y.T.); rami.saade@laumcrh.com (R.S.); sysu@mdanderson.org (S.Y.S.); 4 Department of Otolaryngology–Head and Neck Surgery, ASST Lariana, Ospedale Sant’Anna, University of Insubria, Via Ravona, 21100 Como, Italy; tzmario@inwind.it; 5 Unit of Otorhinolaryngology—Head and Neck Surgery, Department of Biotechnology and Life Sciences, University of Insubria, 21100 Varese, Italy; paolo.castelnuovo@uninsubria.it; 6 Unit of Otorhinolaryngology—Head and Neck Surgery, Department of Medical and Surgical Specialties, Radiologic Sciences, and Public Health, University of Brescia, 25121 Brescia, Italy; davide.mattavelli@unibs.it (D.M.); william.vermi@unibs.it (W.V.); davinter@libero.it (D.L.); 7 Cancer Research United Kingdom & UCL Cancer Trials Centre, University College London, London WC1E 6BT, UK; nicholas.counsell@ucl.ac.uk; 8 Section of Otorhinolaryngology—Head and Neck Surgery, Department of Neuroscience (DNS), University of Padova, 35122 Padova, Italy; marco.ferrari@unipd.it (M.F.); piero.nicolai@unipd.it (P.N.); 9 Department of Otorhinolaryngology—Head and Neck Surgery, University of Brescia, 25121 Brescia, Italy; vittorio.rampinelli@unibs.it; 10 Pathology Unit, Department of Molecular and Translational Medicine, University of Brescia and ASST Spedali Civili di Brescia, 25123 Brescia, Italy; fabio.facchetti@unibs.it; 11 Department of Otolaryngology–Head & Neck Surgery Divisions, Stanford University School of Medicine, Palo Alto, CA 94305, USA; kwpark@stanford.edu (K.W.P.); ahmed.mohyeldin@uci.edu (A.M.); drjfm@stanford.edu (J.F.-M.); 12 Department of Otorhinolaryngology, Head and Neck Surgery, Medical University of Innsbruck, 6020 Innsbruck, Austria; volker.schartinger@i-med.ac.at; 13 Department of Translational Research, University of Pisa, 56126 Pisa, Italy; alessandro.franchi@unipi.it; 14 Unit of Pathology, ASST Sette Laghi, University of Insubria, 21100 Varese, Italy; carla.facco@asst-settelaghi.it (C.F.); fausto.sessa@uninsubria.it (F.S.); roberta.maragliano@asst-settelaghi.it (R.M.); 15 Anatomy and Pathological Histology Section, Department of Molecular and Translational Medicine, University of Brescia, Viale Europa 11, 25123 Brescia, Italy; simonetta.battocchio@gmail.com; 16 Department of Otorhinolaryngology, Head and Neck Surgery and Audiology, Copenhagen University Hospital—Rigshospitalet, 2100 Copenhagen, Denmark; patrick.rene.gerhard.eriksen@regionh.dk (P.R.G.E.); simone.kloch.bendtsen@regionh.dk (S.K.B.); kathrine.kronberg.jakobsen@regionh.dk (K.K.J.); mohamed.elhaddouchi@regionh.dk (M.e.H.); christian.von.buchwald@regionh.dk (C.v.B.); 17 Department of Pathology, Copenhagen University Hospital—Rigshospitalet, 2100 Copenhagen, Denmark; giedrius.lelkaitis@regionh.dk; 18 Department of Otolaryngology—Head and Neck Surgery, Johns Hopkins University School of Medicine, Baltimore, MD 21205, USA; asarasw2@jhmi.edu (A.S.); ramanpreetkaur@jhmi.edu (R.P.K.); mydlarz@jhmi.edu (W.K.M.); mramana3@jhmi.edu (M.R.J.); mishii3@jhmi.edu (M.I.); nlondon2@jhmi.edu (N.R.L.J.); 19 Division of Dentistry, University of Manchester, Manchester M15 6FH, UK; manas.dave@manchester.ac.uk; 20 Faculty of Medicine, School of Cancer Sciences, Cancer Research UK Centre, University of Southampton, Southampton G4 0SF, UK; t.r.fenton@soton.ac.uk; 21 Department of ENT, Glasgow Royal Infirmary, Glasgow G4 0SF, UK; alison.lim3@nhs.scot (A.L.); saleh.okhovat@googlemail.com (S.O.); 22 Homerton Healthcare NHS Foundation Trust, London E9 6SR, UK; gyleen.elegio@nhs.net; 23 Department of Radiation Oncology, Bordeaux University Hospital, REFCOR, 33000 Bordeaux, France; charles.dupin@chu-bordeaux.fr (C.D.); pierre.pouvreau@chu-bordeaux.fr (P.P.); 24 Department of Radiation Oncology, François Baclesse Center, REFCOR, 14076 Caen, France; jthariat@hotmail.com; 25 Department of Oncology, Bordeaux University Hospital, REFCOR, 33000 Bordeaux, France; laurence.digue@chu-bordeaux.fr; 26 Department of Head and Neck Oncology, Institut Gustave Roussy, REFCOR, 94805 Villejuif, France; francoisregis.ferrand@gustaveroussy.fr (F.-R.F.); heloise.de-kermadec@gustaveroussy.fr (H.D.K.); antoine.moya-plana@gustaveroussy.fr (A.M.-P.); 27 Department of Pathology, Montpellier University Hospital, REFCOR, 34295 Montpellier, France; v-costes_martineau@chu-montpellier.fr; 28 Department of Pathology, Bordeaux University Hospital, REFCOR, 33000 Bordeaux, France; claire.castain@chu-bordeaux.fr; 29 Beaumont Hospital, Royal College of Surgeons in Ireland, D09 V2N0 Dublin, Ireland; hintzej@tcd.ie (J.H.); jamespaul.oneill@healthmail.ie (J.P.O.); placy@beaumont.ie (P.L.); woodsr@tcd.ie (R.W.); 30 Royal National Ear, Nose and Throat Hospital, University College London Hospitals NHS Trust, London WC1E 6DG, UK; vazfm@hotmail.com (F.M.V.); paul.o’flynn@nhs.net (P.O.); davidjhoward10@gmail.com (D.J.H.); p.stimpson@ucl.ac.uk (P.S.); v.lund@ucl.ac.uk (V.J.L.); 31 Department of Oncology, Haematology and Bone Marrow Transplantation with Section Pneumology, Hubertus Wald Tumorzentrum, University Medical Center Hamburg-Eppendorf, 20246 Hamburg, Germany; s.wang@uke.de; 32 Department of Histopathology, University College London Hospitals NHS Trust, London WC1E 6JA, UK; amritajk@msn.com; 33 Head and Neck Centre, University College London Hospitals NHS Trust, London NW1 2BU, UK; dawn.carnell@nhs.net; 34 Department of Clinical Oncology, The Christie NHS Foundation Trust, Manchester M20 4BX, UK; david.thomson2@nhs.net; 35 Department of Otorhinolaryngology and Head and Neck Surgery, Central University Hospital of Asturias, 33011 Oviedo, Spain; jllorente@uniovi.es; 36 Department of Head and Neck Oncology, Instituto de Investigación Sanitaria del Principado de Asturias, 33011 Oviedo, Spain; mariohermsen@gmail.com; 37 Institute of Pathology, Ludwig Maximilians Universität, 80337 Munich, Germany; philipp.jurmeister@med.uni-muenchen.de; 38 Department of Neuropathology, Charité—Universitätsmedizin, 10117 Berlin, Germany; david.capper@charite.de; 39 Department of Neurosurgery, Johns Hopkins University School of Medicine, Baltimore, MD 21287, USA; ggallia1@jhmi.edu; 40 Department of Otolaryngology-Head & Neck Surgery, National University of Singapore, Singapore 119228, Singapore; joshtay@nus.edu.sg; 41 Department of Radiation Oncology, Stanford University School of Medicine, Palo Alto, CA 94305, USA; qle@stanford.edu; 42 Department of Pathology, Stanford University School of Medicine, Palo Alto, CA 94305, USA; rbwest@stanford.edu; 43 Rhinology and Endoscopic Skull Base Surgery, Department of Otolaryngology—Head and Neck Surgery, Stanford University School of Medicine, Palo Alto, CA 94305, USA; zmpatel@stanford.edu (Z.M.P.); jnayak@stanford.edu (J.V.N.); hwangph@stanford.edu (P.H.H.); 44 Department of Medical Oncology, The University of Texas MD Anderson Cancer Center, Houston, TX 77030, USA; rferrarotto@mdanderson.org; 45 Department of Radiation Oncology, The University of Texas MD Anderson Cancer Center, Houston, TX 77030, USA; jphan@mdanderson.org; 46 Department of Biomedical Sciences, Humanitas University, Via Rita Levi Montalcini 4, Pieve Emanuele, 20072 Milan, Italy; paolo.bossi@hunimed.eu; 47 IRCCS Humanitas Research Hospital, via Manzoni 56, Rozzano, 20089 Milan, Italy; 48 Unit of Otorhinolaryngology, IRCCS IEO, 20141 Milano, Italy; 49 Division of Otolaryngology and Head and Neck Surgery, Université Paris Saclay, 78 Rue du Général Leclerc, Le Kremlin-Bicêtre, 94270 Paris, France; 50 Department of Head and Neck Surgery, Hôpital Lariboisière, Assistance Publique Hôpitaux de Paris, Université Paris Cité, REFCOR, 75475 Paris, France; benjamin.verillaud@aphp.fr; 51 Department of Integrative Oncology, British Columbia Cancer Research Center, Office 6-112, Vancouver, BC V5Z 1L3, Canada; cgarnis@bccrc.ca; 52 Division of Otolaryngology and Head and Neck Surgery, University of British Columbia, Vancouver, BC V5Z 1M9, Canada; andrew.thamboo@gmail.com; 53 Department of Otolaryngology-Head and Neck Surgery, Mayo Clinic, Rochester, MN 55902, USA; felicia.o.olawuni@gmail.com (F.O.); moore.eric@mayo.edu (E.J.M.); 54 Department of Otolaryngology-Head and Neck Surgery, University of Pittsburgh Medical Center, Pittsburgh, PA 15213, USA; chobygw2@upmc.edu; 55 Department of Otolaryngology—Head and Neck Surgery, Mayo Clinic, Phoenix, AZ 85054, USA; lal.devyani@mayo.edu; 56 Department of Thoracic/Head and Neck Medical Oncology, The University of Texas MD Anderson Cancer Center, Houston, TX 77030, USA; nakhave@mdanderson.org; 57 Department of Pathology, University of Pittsburgh, Pittsburgh, PA 15261, USA; belldiana@yahoo.com; 58 Sinonasal and Skull Base Tumor Section, Surgical Oncology Program, Center for Cancer Research, National Cancer Institute, National Institutes of Health, Bethesda, MD 20892, USA

**Keywords:** sinonasal undifferentiated carcinoma, SNUC, survival outcomes

## Abstract

**Background:** Sinonasal undifferentiated carcinoma (SNUC) is an extremely rare, high-grade, and aggressive tumor of the sinonasal tract. Due to the rarity of this malignancy, current treatment guidelines are based on small and often/mainly single-center retrospective datasets. In the absence of a universally accepted standard of care for SNUC, treatment approaches vary across countries and institutions, reflecting real-world clinical practice. The primary aim of this study was to describe real-world treatment and outcomes for patients with confirmed SNUC. **Methods:** This was an international, multi-center, retrospective, observational cohort study that pooled patients into the largest SNUC dataset to date. Fifteen centers were enrolled to contribute data, including seven from Europe, four from the United States, three from the United Kingdom, and one from Canada. In the absence of a universally accepted standard of care for SNUC, treatment approaches varied across countries and institutions, reflecting real-world clinical practice. Patients included were those with histologically confirmed SNUC who were treated between 1997 and 2021. **Results:** This study yielded 485 patients treated for SNUC. The median age at diagnosis was 55.6 years (IQR: 44.5–67.6), and 63.7% were male. Most cases presented at advanced stages, with 70.8% as T4a or T4b. Overall survival (OS) outcomes were available for 412 patients, with a median follow-up of 26.0 months. The 5- and 10-year OS were 47.2% (95% CI: 40.8–53.3%) and 39.6% (95% CI: 32.5–46.6%), respectively. Advanced age, dichotomized T-stage (T4a/b vs. T1–3), M-stage, and orbital involvement were significant poor prognostic factors on univariable analysis (*p*’s < 0.01). On multivariable analysis, orbital involvement (HR: 2.73, 95% CI: 1.42–5.27, *p* = 0.003) and distance metastasis stage (HR: 3.00, 95% CI: 1.25–7.21, *p* = 0.014) were both independently associated with worse OS. **Conclusions:** This observational study presents the largest multi-center cohort analysis of SNUC to date, providing new insights into prognostic factors for a rare cancer treated at global centers of excellence. Orbital involvement and the presence of metastases are candidate independent risk factors associated with poorer OS.

## Simple Summary

Sinonasal undifferentiated carcinoma (SNUC) is a very rare and aggressive cancer that arises from the nasal cavity and paranasal sinuses. Due to its rarity, there are no established standards of treatment. Patients often present with a disease that has advanced into adjacent organs such as the eye and the brain, as well as distant spread into organs such as the liver or lung. This study collected information from 485 patients from multiple countries across three continents to examine treatment paradigms in several global centers of excellence, studying factors that may influence patient survival. This study found that involvement of the orbit and the presence of distant spread were associated with worse outcomes, and found that traditional tumor classification and staging measures were less predictive. These findings provide valuable insights into the need for redefining a staging system that may more accurately prognosticate and stratify treatment escalation strategies.

## 1. Introduction

Sinonasal undifferentiated carcinoma (SNUC) is an extremely rare, high-grade, and aggressive tumor of the sinonasal tract with an age-adjusted incidence of 0.02 per 100,000 people per year in the United States [1]. SNUC is a diagnosis of exclusion, though it demonstrates some histological and morphological similarity to other sinonasal neuroendocrine tumors, such as olfactory neuroblastoma and neuroendocrine carcinoma. Additionally, immunohistochemical and molecular evaluation for NUT, SMARCB1, and SMARCA4 alterations is essential to exclude these important differential diagnoses [1,2,3,4,5,6,7,8,9].

Symptoms of SNUC initially overlap with those of benign sinonasal conditions, delaying diagnosis, and when combined with the aggressive nature of SNUC, patients typically present at an advanced stage, characterized by extensive local invasion, as well as locoregional and distant metastases compared to other sinonasal malignancies [2,3]. As such, 5-year overall survival for SNUC is usually below 50%, with reports as low as 20% [4,5,6].

Due to the rarity of this malignancy, guidelines are based on data from single-center series or meta-analyses of public databases. Standard of care in most centers of excellence includes multimodal management of disease; numerous studies report superior efficacy of a multimodal approach compared to single modality treatment [2,4,7,8,9]. There are two primary treatment paradigms. The first utilizes induction chemotherapy to assess treatment response, followed by definitive chemoradiation in responders with or without salvage surgery. Induction chemotherapy has been shown to be useful in selecting likely responders for definitive chemoradiation [3,10].

The second paradigm starts with surgery with adjuvant radiation/chemoradiation [10]. Due to extra sinus involvement (brain, orbit) that is typical at presentation, negative margins are difficult to achieve at surgical resection and, therefore, chemoradiation +/− prior induction chemotherapy is usually offered.

Due to the rarity of this malignancy, guidelines are based on data from small case series or meta-analyses of public databases. Multi-institutional real-world studies of rare malignancies, such as SNUC, offer a pragmatic way to study outcomes and potentially offer best practices yet to be published that might inform and optimize outcomes for patients. This novel multi-center study retrospectively analyzes data from 15 centers on histologically confirmed SNUC treated between 1997 and 2021.

## 2. Materials and Methods

### 2.1. Patients

We conducted a retrospective review of clinical data for patients treated between 1997 and 2021 from 15 international centers: The University of Texas MD Anderson Cancer Center (Houston, TX, USA), the Johns Hopkins University School of Medicine (Baltimore, MD, USA), the Mayo Clinic (Rochester, NY, USA and Phoenix, AZ, USA), Stanford University School of Medicine (Palo Alto, CA, USA), University of Manchester (Manchester, UK), University of British Columbia (Vancouver, BC, Canada), University of Copenhagen (Copenhagen, Denmark), Beaumont Hospital (Dublin, Ireland), Ludwig Maximilians University Hospital Munich (Munich, Germany), University College London (London, UK), Central University Hospital of Asturias (Oviedo, Spain), University of Insubria (Varese, Italy), Glasgow Royal Infirmary (Glasgow, Scotland, UK), Università degli Studi di Brescia-ASST Spedali Civili di Brtescia (Brescia, Italy), and REFCOR (French Network of Rare Head and Neck Tumors) (France).

Data collected include patient demographics (age and sex), disease characteristics including tumor classification and stage, treatment details, and survival outcomes. IRB approval was obtained from all institutions, with further approval for multi-center data analysis from University College London IRB/Research Ethics Committee (UCL REC no. 9609/002).

### 2.2. Diagnoses and Treatment of SNUC

The date of diagnosis was defined as the date of biopsy. In all patients, the histopathological diagnosis was conducted by pathologists with expertise in the field. Patients were treated by their respective institution’s clinical practice, and all institutions involved are tertiary-level centers of excellence with longstanding experience in the diagnosis and management of this disease.

### 2.3. Statistical Analysis and Clinical Data

The primary aim of this study was to describe the current real-world clinical practice and investigate clinical outcomes in patients with confirmed SNUC. The primary endpoint is overall survival (OS), calculated from the date of diagnosis and censored at the date last known to be alive. OS was defined using the Kaplan–Meier method and log-rank tests. Univariable and Multivariable Cox proportional hazards regression analyses were performed to estimate hazard ratios (HRs), 95% confidence intervals (CIs), and corresponding *p*-values.

Multivariable analysis was conducted in two stages to evaluate prognostic and treatment-related factors while appropriately adjusting for potential confounders (where available). This modeling approach was chosen to be able to assess the impact of baseline prognostic factors as well as treatment effects, whilst allowing for the limitations of missing data in the sample.

Stage 1—Identification of Prognostic Covariates:

Univariable Cox proportional hazards regression was first used to explore the associations between baseline clinical and pathological characteristics and OS. Variables with a significance level of *p* < 0.05 were selected for inclusion in a multivariable model to assess independent prognostic factors of OS.

Stage 2—Evaluation of Treatment Effects:

Post-diagnosis treatment-related variables were evaluated for associations with OS both in the univariable setting and then in the multivariable setting, adjusting for the independent prognostic factors identified in Stage 1.

Analyses were conducted using available-case data, and sample sizes for each variable were reported. While multiple imputation was considered, the small sample size and high proportion of missing data for key variables meant that imputation could introduce bias and offer limited improvement; therefore, we proceeded with available-case analyses.

## 3. Results

### 3.1. Patient Characteristics

A total of 485 patients with SNUC were included. 63.7% (*n* = 309) were male, and the median age at diagnosis was 55.6 years (IQR: 45.5–67.0). Two-thirds of cases were T4a or T4b (70.8%, 206/291), and most did not exhibit nodal involvement (80.3% N0, 147/183). Distant metastasis at presentation was rare (2.7%, 7/261). Almost half of the tumors involved the paranasal sinuses (48.0%, 233/412), with the ethmoid sinuses being the most commonly involved (58.9%, 109/185). Dural and orbital invasion were present in 59.9% (118/197) and 49.6% (127/256) of cases, respectively (Table 1).

### 3.2. Patient Outcomes and Prognostic Factors

**Figure 1:**
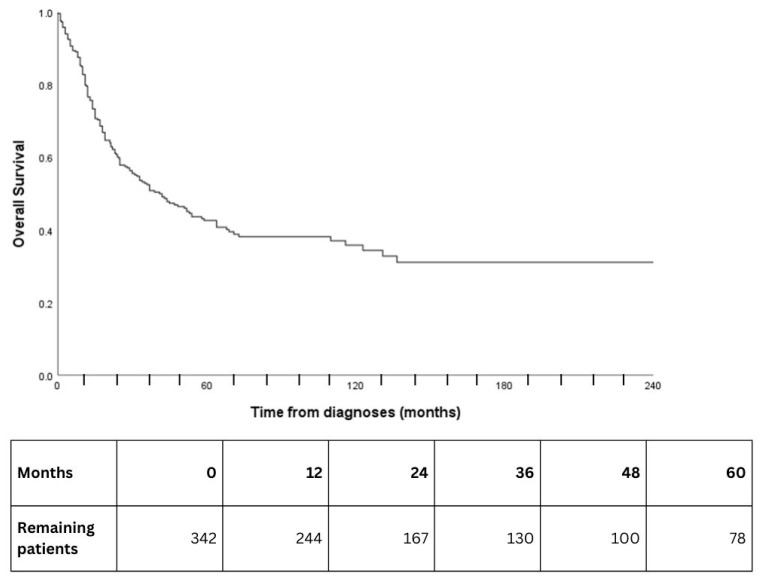
Overall survival.

After a median follow-up of 26.0 months (IQR: 11–63 months), 43.4% (179/412) of patients had died. The 1-, 3-, 5- and, 10-year OS rates were 77.1% (95% CI: 72.0–81.4%), 55.3% (95% CI: 49.2–61.0%), 47.2% (95% CI: 40.8–53.3%), and 39.6% (95% CI: 32.5–46.6%), respectively (Figure 1). Overall, 37.8% (*n* = 129/341) of patients relapsed with a median time to recurrence of 12.0 months (IQR: 7.0–19.0). Distant recurrences were frequent (42.4%, 39/92), and local recurrences occurred in 26.1% (24/92) of cases. A total of 20.7% (19/92) of patients experienced local, regional, and distant recurrence.

**Figure 2:**
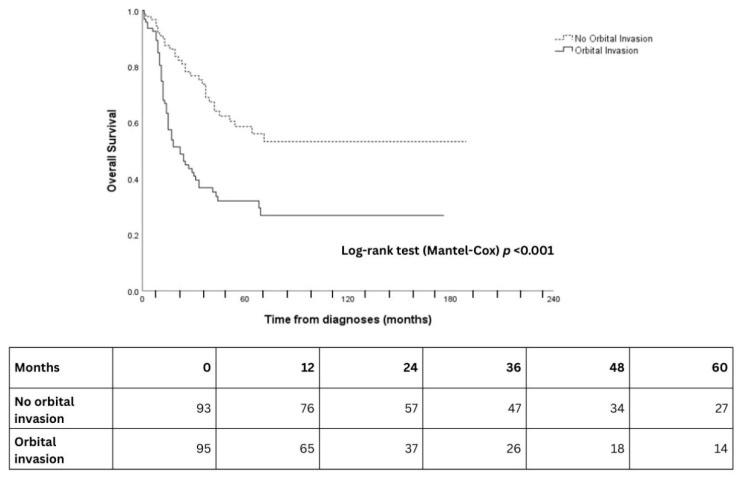
Kaplan–Meier curves for overall survival according to orbital invasion at presentation. Solid line = orbital invasion at presentation; dashed line = no orbital invasion at presentation. Numbers at risk at 12-month intervals are shown below the x-axis.

**Figure 3:**
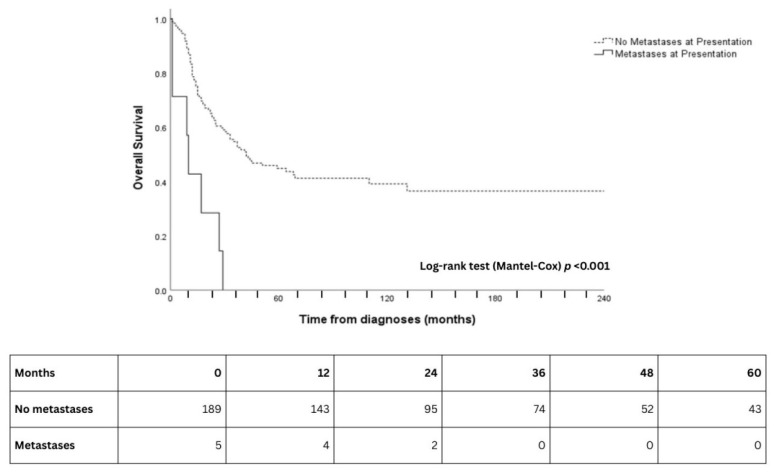
Kaplan–Meier curves for overall survival according to metastases at presentation. Solid line = metastases at presentation; dashed line = no metastases at presentation. Numbers at risk at 12-month intervals are shown below the x-axis.

With univariable analysis, age, dichotomized tumor classification (T4 vs. T1–3), presence of metastases, and orbital involvement were significantly associated with prognosis as it pertains to overall survival (*p* < 0.05; Table 2). These variables were subsequently included in the primary multivariable model. Orbital involvement (HR: 2.73, 95% CI: 1.42–5.27, *p* = 0.003) (Table 2, Figure 2), M-classification (HR: 3.00, 95% CI: 1.25–7.21, *p* = 0.014), and age (HR: 1.02, CI: 1.01–1.04, *p* = 0.028) remained independent prognostic variables on multivariable analysis (Table 2, Figure 3). There was almost a two-fold increase in risk for T4 compared to T1-3, although this did not reach statistical significance in the smaller sample size (HR: 1.72, 95% CI: 0.77–3.83, *p* = 0.184).

### 3.3. Treatment-Based Outcomes

Treatment information was accessible for 330 patients (Table 1). Surgery was reported in 50.6% of cases with available data (167/330), induction chemotherapy in 54.1% (132/244), and adjuvant chemotherapy in 62.7% (141/225). Adjuvant radiotherapy was administered in 85.3% of cases (192/225). Immunotherapy and neoadjuvant chemotherapy or chemoradiotherapy (alongside surgery) were less frequently used, reported in 4.6% (7/152) and 32.2% (46/143) of patients, respectively.

Among 210 patients with available data on radiation modality, Intensity-Modulated Radiation Therapy (IMRT) was the most common (134/210, 63.8%), followed by proton therapy (36/210, 17.1%), 3D conformal radiotherapy (29/210, 13.8%), radiotherapy combined with Stereotactic Radiosurgery (SRS) (7/210, 3.3%), and 2D radiotherapy (4/210, 1.9%). Radiation dose information was available for 223 patients, of whom 162/223 (72.6%) received >60 Gy and 61/223 (27.4%) received ≤60 Gy.

Univariable analysis included IMRT versus 3D radiotherapy (HR: 0.40, 95% CI: 0.24–0.66, *p* < 0.001) and proton therapy versus 3D radiotherapy (HR: 0.22, 95% CI: 0.08–0.59, *p* = 0.003), whilst receiving surgery was associated with worsening survival (HR: 0.22, 95% CI: 0.08–0.59, *p* = 0.003). In multivariable analysis, adjusting for independent baseline prognostic factors by including orbital involvement and presence of metastases in each model, treatment effects remained in the same direction, but none reached statistical significance due to the reduction in sample size due to missing data (Table 3).

**Figure 4:**
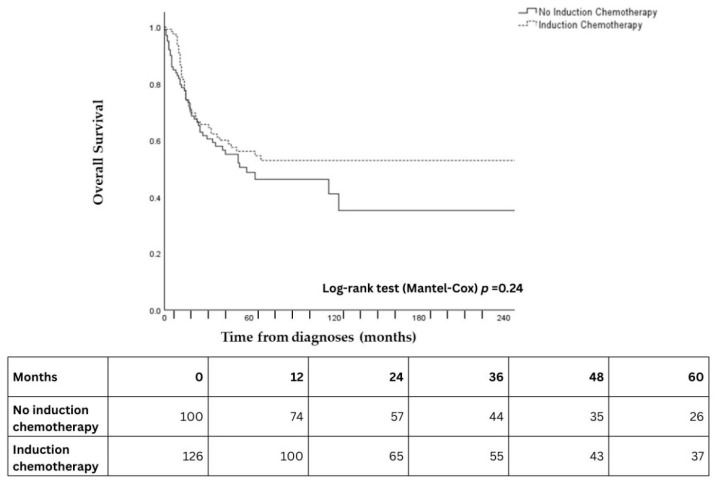
Kaplan–Meier curves for overall survival according to induction chemotherapy status. Solid line = no induction chemotherapy; dashed line = induction chemotherapy. Numbers at risk at 12-month intervals are shown below the x-axis.

Of the 485 patients included in the study, 228 had available data on both induction chemotherapy (IC) and overall survival. The 5-year OS rate was 54.6% (95% CI: 44.6–64.6%) in patients who received IC compared to 48.7% (95% CI: 37.7–59.7%) in those who did not (HR = 0.79, 95% CI: 0.53–1.17, *p* = 0.24) (Figure 4).

## 4. Discussion

Here, we present clinical data from 485 primary cases of SNUC with follow-up information for 412 patients, representing the largest multi-center study reported to date. The 5-year overall survival in this cohort was 47.2%, which is consistent with previous reports, including a recent case series of 40 patients reported by Gamez et al. [4]. However, survival rates have been reported to range from as high as 74% to as low as 20%, suggesting the high heterogeneity of this tumor type and the need for molecular characterization [4,5,6,7,8,9,10,11,12,13,14].

Orbital involvement was present in about half of the patients. This largely aligns with recently published studies [2,15]. Crucially, of the clinicopathological factors assessed in the present study, orbital involvement was most significant and substantially detrimental to overall survival. Indeed, the impact of T-classification was reduced after adjusting for orbital involvement and metastatic disease, which aligns with a recent meta-analysis, where no difference in cumulative overall survival was observed between low- and high-tumor classification malignancy [16]. Orbital involvement and the presence of metastases were independent risk factors associated with poorer outcomes, but T and N classification did not show a strong association in multivariable analysis. This suggests that orbital involvement may possibly be a more significant prognostic factor than traditional TNM classification and overall stage. Saraswathula et al. demonstrated comparable rates of orbital involvement at presentation, aligning with our findings and reflecting the aggressive nature of SNUC [17]. Although Saraswathula et al. showed that orbital extension of recurrent/metastatic SNUC was not associated with prognosis, it was, however, linked to a higher probability of recurrent disease following treatment [17]. Therefore, there may be a role for more aggressive treatment modalities in this subgroup of patients with orbital involvement. Orbital involvement should be considered an important factor in the treatment planning of SNUC. The data supports that orbital involvement represents an important factor.

After adjusting for baseline prognostic factors, no treatment modality was found to offer superior survival outcomes, with strong evidence given the small sample size available for multivariable analysis of treatment effects. However, receiving adjuvant radiotherapy approximately halved the risk, but with a wide confidence interval; similarly, modern radiotherapy techniques may be associated with longer survival. Data also suggested that surgery may lead to an increased risk. The role of aggressive surgery, such as orbital exenteration, remains controversial [17], with some studies demonstrating limited utility and significant associated morbidity [18,19,20,21,22,23], while others show positive effects on overall survival and recurrence-free survival. Therefore, further research is needed to aid in clinical decision-making [21].

In recent years, there has been a trend toward the use of definitive chemoradiation with or without induction chemotherapy instead of surgery as a first-line treatment. This may be due to the extensive local invasion of the tumor that renders the attainment of negative margins infeasible, as well as the morbidity associated with aggressive surgical approaches. Studies suggest that outcomes with the two approaches are comparable, although the evidence remains limited [8]. In the present study, there was no strong evidence of a difference in survival outcomes for these two approaches. This implies that definitive chemoradiation may be more suitable, particularly for surgically complex cases. Indeed, in a recent study of the French Network of Rare Head and Neck Cancers (REFCOR) cohort, surgery did not appear to lead to better outcomes [15].

Regarding induction chemotherapy prior to definitive chemoradiation, Amit et al. and London et al. have previously demonstrated efficacy prior to definitive treatment [3,10]. Of note, IC has been shown to enable the selection of patients who are likely to respond to definitive chemoradiotherapy [10]. The role of induction chemotherapy as treatment selection for further locoregional therapy may also offer a tailored approach, giving the possibility to increase ocular preservation and to offer survival benefits [24]. In a study of forty-two sinonasal cancer patients, induction chemotherapy was shown to be prognostic of overall response to treatment [24]. Furthermore, Takahashi et al. identified thirty-four differentially expressed genes, which may further identify patients who are more likely to respond [24]. In contrast, the latest analysis of the REFCOR cohort and similar studies have not demonstrated differences in survival outcomes with the addition of induction chemotherapy in any setting [15,25]. This was similarly observed in a recent analysis of the National Cancer Database [20,22].

Alternative treatment strategies may involve targeted treatment specific to SNUC subtypes [17]. Isocitrate dehydrogenase 2 (IDH2) mutations are found in subtypes of SNUC, with ongoing trials evaluating the efficacy of IDH2-targeted agents, which may clarify their clinical utility and scope [23]. Further studies will be needed to determine how these agents may be used to complement current treatment modalities. Additionally, combined immunotherapy strategies, such as anti-programmed death-ligand agents and IL-15-stimulated interferon gamma release from NK cells, have demonstrated a 9.6-fold enhancement in natural killer cell-mediated killing of SNUC cells in pre-clinical models [22]. However, these approaches require further investigation beyond pre-clinical trials.

## 5. Limitations

We acknowledge several limitations in this study. First, its retrospective observational design introduces the possibility of selection bias and residual confounding. As with all non-randomized cohort studies, the ability to draw causal inferences is limited, and associations observed may be influenced by unmeasured or uncontrolled confounders. Second, although the multi-center design allowed for a broader representation of practice, it also introduced variability in clinical reporting, diagnosis, and treatment approaches. Despite considerable efforts to standardize data collection and ensure consistency, inter-center differences were unavoidable. Propensity score adjustment was not possible owing to missing data in critical treatment and baseline variables, precluding reliable estimation of treatment assignment. In addition, analysis of disease-free survival and recurrence-free survival was not feasible because recurrence data and dates were inconsistently reported across centers, precluding reliable time-to-event analyses for these outcomes. Moreover, the retrospective, multi-center nature of this study limited data completeness. Surgical procedures were not uniformly sub-classified into primary, neoadjuvant, or salvage settings, and all surgical cases are presented collectively. The same also prevented stratified analyses of induction chemotherapy, followed by definitive treatment. These factors may impact the interpretation of treatment-based outcomes.

Furthermore, a significant limitation here, which is common in such studies, is the extent of missing data. While we aimed to include all relevant variables in multivariable analysis, this led to a substantial reduction in sample size. Incomplete and non-overlapping availability of TNM staging and orbital involvement data across centers precluded the development of an updated TNM classification system. Nevertheless, this study provides a template of essential clinical, radiologic, and molecular variables to guide future work in rare sinonasal malignancies and underscores the importance of structured, prospective data collection for refining staging and prognostic models. Nevertheless, our study is the largest cohort of this rare disease reported to date, so associations can be estimated with reasonable precision, and the robustness of these effects has been explored in adjusted models. It is possible that data were not missing at random; missingness may have been related to key clinical factors or outcomes, which could introduce bias and limit the generalizability and reliability of the results. Also, a temporal analysis could not be performed due to incomplete or inconsistent documentation of treatment dates across participating centers, limiting our ability to assess changes in clinical practice or outcomes over time. This is relevant given the evolving treatment landscape for SNUC, as we still observe varying treatment approaches between institutions and countries. Finally, this study did not incorporate molecular profiling. Recent evidence suggests that SNUCs consist of biologically distinct molecular subgroups with different prognoses and potentially varying treatment responses [26,27,28]. Integration of molecular data was beyond the scope of this study but represents an important area for future research. Studies that link clinical outcomes with molecular characteristics may help clarify whether treatment effectiveness differs across subgroups.

## 6. Conclusions

In conclusion, this real-world observational study represents the largest international collaboration and multi-center multinational analysis of SNUC to date, providing valuable insights into prognostic factors, treatment approaches, and survival outcomes. Orbital involvement emerged as a strong independent prognostic factor for overall survival, outperforming traditional TNM classification and stage in prognostic value. These findings highlight the need to incorporate additional parameters beyond traditional TNM classification and stage to more accurately inform patient prognosis and guide treatment strategies in SNUC. Additionally, more extensive treatment beyond traditional strategies may be warranted in cases with orbital involvement. Further research is needed to evaluate the potential role of targeted treatments in these patients.

## Figures and Tables

**Table 1:** Frequency of clinical characteristics at presentation.

Clinical Characteristic		No. (%) of Total Patients	Patients with Survival Data Available, No. (%)
Age, Years	Median (IQR)	55.6 years (45.5–67.0)	55.6 years (45.5–67.6)
Gender	Male	309 (63.7%)	263 (63.8%)
	Female	176 (36.3%)	149 (36.2%)
T-classification	T1	22 (7.6%)	9 (3.4%)
	T2	32 (11.4%)	27 (10.2%)
	T3	31 (10.7%)	30 (11.3%)
	T4a/T4b	206 (70.8%)	200 (75.2%)
N-classification	N0	147 (80.3%)	142 (80.2%)
	N1 or Greater	36 (19.7%)	35 (19.8%)
M-classification	M0	254 (97.3%)	248 (97.3%)
	M1	7 (3.8%)	7 (2.7%)
Sinus Involvement	Nasal Cavity Only	179 (43.4%)	168 (43.0%)
	Sinus Involvement	233 (56.6%)	223 (57.0%)
Sinus Affected	Ethmoid	109 (58.9%)	108 (59.3%)
	Maxillary	49 (26.5%)	48 (26.4%)
	Frontal	11 (5.9%)	10 (5.5%)
	Sphenoid	16 (8.6%)	16 (8.8%)
Neck Disease	Yes	35 (22.4%)	34 (22.2%)
	No	121 (77.6%)	119 (77.8%)
Orbital Involvement	No	129 (50.4%)	123 (50.4%)
	Yes	127 (49.6%)	121 (49.6%)
Dural Invasion	No	79 (40.1%)	75 (41.0%)
	Yes	118 (59.9%)	108 (59.0%)
Underwent Surgery	Yes	167 (50.6%)	160 (50.0%)
	No	163 (49.4%)	160 (50.0%)
Induction Chemotherapy	Yes	132 (54.1%)	127 (54.2%)
	No	112 (45.9%)	107 (45.7%)
Adjuvant Radiotherapy	Yes	192 (85.3%)	106 (77.4%)
	No	33 (14.7%)	31 (22.6%)
Adjuvant Chemotherapy	Yes	141 (62.7%)	138 (63.6%)
	No	84 (37.3%)	79 (36.4%)
Neoadjuvant Chemoradiotherapy/ Radiotherapy	Yes	46 (32.2%)	44 (32.6%)
	No	97 (67.8%)	91 (67.4%)
Immunotherapy	Yes	7 (4.6%)	7 (2.1%)
	No	145 (95.4%)	142 (95.3%)

**Table 2:** Univariable and Multivariable Cox regression overall survival analyses of clinical and tumor characteristics.

	Overall Survival Univariable (*n*)	Overall Survival Univariable (HR, 95% CI, *p*)	Overall Survival Multivariable * (*n*)	Overall Survival Multivariable * (HR, 95% CI, *p*)
Age	314	HR: 1.03, 95% CI: 1.02–1.04, ***p* < 0.001**	158	HR: 1.02, 95% CI: 1.01–1.04, ***p* = 0.028**
Sex (Female versus Male)	318	HR: 0.98, 95% CI: 0.84–1.14, *p* = 0.771	N/A	N/A
Sinus Involvement versus Nasal Cavity Only	297	HR: 1.26, 95% CI: 0.93–1.72, *p* = 0.14	158	N/A
Neck Disease (reference: none)	152	HR: 0.99, 95% CI: 0.55–1.80, *p* = 0.973	N/A	N/A
Orbital Involvement (reference: none)	166	HR: 2.49, 95% CI: 1.62–3.84, ***p* < 0.001**	158	HR: 2.73, 95% CI: 1.42–5.27, ***p* = 0.003**
Dural Invasion (reference: none)	152	HR: 1.18, 95% CI: 0.73–1.92, *p* = 0.501	N/A	N/A
T-classification (<T4 versus T4)	181	HR: 2.21, 95% CI: 1.22–4.00, ***p* = 0.009**	158	HR: 1.720, 95% CI: 0.773–3.82, *p* = 0.184
N-classification (N0 versus ≥N1)	172	HR: 1.04, 95% CI: 0.59–1.82, *p* = 0.901	N/A	N/A
M-classification (M0 versus M1)	197	HR: 4.83, 95% CI: 2.19–10.65, ***p* < 0.001**	158	HR: 3.00, 95% CI: 1.25–7.21, ***p* = 0.014**

* Model: Overall Survival ~ Age + Sinus Involvement + Orbital Involvement + M-Stage + T-Stage. Bold formatting highlights results that are statistically significant (*p* < 0.05).

**Table 3:** Univariable and Multivariable Cox regression overall survival analyses of treatment characteristics.

	Overall Survival Univariable (*n*)	Overall Survival Univariable (HR, 95% CI)	Overall Survival Multivariable * (*n*)	Overall Survival Multivariable * (HR, 95% CI)
Radiation dose (<60 versus >60 Gy)	217	HR: 1.01, 95% CI: 0.64–1.59, *p* = 0.98	N/A	N/A
Surgery (reference: none)	261	HR: 1.24, 95% CI: 0.87–1.77, *p* = 0.236	N/A	N/A
Induction Chemotherapy (reference: none)	228	HR: 0.79, 95% CI: 0.53–1.17, *p* = 0.237	N/A	N/A
Adjuvant Radiotherapy (reference: none)	157	HR: 0.64, 95% CI: 0.39–1.06, *p* = 0.085	N/A	N/A
Adjuvant Chemotherapy (reference: none)	157	HR: 0.91, 95% CI: 0.60–1.38, *p* = 0.67	N/A	N/A
Immunotherapy (reference: none)	148	HR: 0.93, 95% CI: 0.65–1.33, *p* = 0.693	N/A	N/A
IMRT versus 3D	158	HR: 0.40, 95% CI: 0.24–0.66, ***p* < 0.001**	89	HR: 0.65, 95% CI: 0.23–1.87, *p* = 0.424
Proton Beam Therapy versus 3D	61	HR: 0.22, 95% CI: 0.08–0.59, ***p* = 0.003**	37	HR: 0.37, 95% CI: 0.10–1.43, *p* = 0.150
Proton Beam Therapy versus IMRT	162	HR: 0.53, 95% CI: 0.21–1.34, *p* = 0.182	N/A	N/A

* Model: Overall Survival ~ Orbital Involvement + M-Stage + [treatment variable, one-at-a-time]. Bold formatting highlights results that are statistically significant (*p* < 0.05).

## Data Availability

Data is available upon reasonable request to the corresponding authors.

## Abbreviations

The following abbreviations are used in this manuscript:

SNUC	Sinonasal Undifferentiated Carcinoma
OS	Overall Survival
HR	Hazard Ratio
CI	Confidence Interval
IQR	Interquartile Range
IRB	Institutional Review Board
REC	Research Ethics Committee
IC	Induction Chemotherapy
IMRT	Intensity-Modulated Radiation Therapy
SRS	Stereotactic Radiosurgery
TNM	Tumor, Node, Metastasis Staging System
3D	Three-Dimensional (Radiotherapy)
2D	Two-Dimensional (Radiotherapy)
